# Factors Influencing the Healing of Maxillary Sinusitis of Endodontic Origin After Non-Surgical Endodontic Treatment

**DOI:** 10.3390/jcm14196778

**Published:** 2025-09-25

**Authors:** Paweł Szczurowski, Krzysztof Gronkiewicz, Barbara Czopik

**Affiliations:** 1 Department of Cranio-Maxillo-Facial Surgery, Oncology and Reconstructive Surgery, Jagiellonian University Medical College, 30-688 Kraków, Poland; pawel.szczurowski@uj.edu.pl; 2 Department of Dental Prosthetics and Orthodontics, Jagiellonian University Medical College, 31-155 Kraków, Poland; krzysztof.gronkiewicz@uj.edu.pl; 3 Private Endodontic Practice PS24, 30-654 Kraków, Poland

**Keywords:** endodontic treatment, maxillary sinusitis of endodontic origin, MSEO, root canal treatment, maxillary sinusitis of dental origin, MSDO

## Abstract

**Background/Objectives**: The purpose of this study was to indicate factors influencing the healing of maxillary sinusitis of endodontic origin (MSEO) after non-surgical endodontic treatment. **Methods**: The study was performed retrospectively on medical records and CBCT data of 114 teeth in 114 patients, who were referred to endodontic treatment between 2016 and 2024, performed by the same operator and according to the same treatment protocol. Fifteen factors were chosen for their possible influence on the healing of MSEO. **Results**: The rate of the complete healing of MSEO after RCT was 76.32%. The healing of MSEO was higher when CHX was applied in the final irrigation protocol (*p* = 0.022) and was less likely when there was a flare-up in-between visits or after obturation of the canals (*p* = 0.002). MSEO was more likely to heal when a tooth was treated in two appointments than with single-visit RCT (*p* = 0.012). The number of endodontic interventions significantly influenced the healing of MSEO, as it was less likely to heal when there was more than one endodontic retreatment for a tooth (*p* = 0.01). **Conclusions**: Within the limitations of this retrospective study, four factors significantly influenced the healing of MSEO, and these should be taken into consideration in obtaining treatment protocols for dental-induced sinusitis and the better assessment of the possible success of this non-invasive treatment approach.

## 1. Introduction

The etiopathogenesis of maxillary sinusitis of dental origin (MSDO) is well known, as 40–70% of unilateral cases of sinusitis can be induced by an odontogenic cause [1,2]. In 2018, the American Association of Endodontists (AAE) excluded maxillary sinusitis of endodontic origin (MSEO) from the group of dental-induced sinusitis to emphasize the role of endodontic infection as an important cause in MSDO development [3]. Chronic apical periodontitis is the second most common cause of all MSDO incidents [4,5], yet there are not many sources of information for the best management options and treatment regimens that include the involvement of endodontic consultation and treatment [6]. Moreover, the literature presents no common treatment protocols for maxillary sinusitis of endodontic origin. Very few studies address the impact of root canal treatment (RCT) on odontogenic sinusitis, presenting that root canal treatment may be useful as a pre-treatment for endoscopic sinus surgery (ESS) procedures or effective as a single-mode treatment for the complete resolution of sinusitis symptoms [7,8].

Odontogenic maxillary sinusitis has a unique microbiology that is similar on both dental and sinonasal sites [9]. The fact that the same microbiota were isolated from inflamed maxillary sinuses and periapical abscesses of adjacent teeth confirms the correlation between endodontic infection and unilateral maxillary sinusitis [9,10,11]. This emphasizes the role of non-surgical endodontic treatment as the one that should be taken into consideration as a therapeutic option when MSEO is suspected [11].

The purpose of this study was to indicate factors influencing the healing of maxillary sinusitis of endodontic origin after non-surgical endodontic treatment and therefore contribute to obtaining treatment protocols for dental-induced sinusitis and the better assessment of the possible success of this non-invasive treatment approach.

## 2. Materials and Methods

### 2.1. Study Group

The study was performed retrospectively on medical records and cone beam computed tomography (CBCT) data obtained from patients, who were referred to endodontic treatment between 2016 and 2024. A total number of 397 teeth in 310 patients initially qualified for the study; however, due to strict inclusion and exclusion criteria, 114 teeth in 114 patients finally entered the study.

### 2.2. Inclusion Criteria (All of Following)

- Age > 18.
- No systemic diseases (ASA score = 1).
- Maxillary sinusitis associated with either premolar or molar apical periodontitis confirmed with CBCT scans.
- PAO and/or PAM signs in CBCT scans before the treatment.
- Teeth that underwent non-surgical RCT performed by the same operator (BC) according to a defined treatment protocol.
- Asymptomatic or symptomatic cases with either dental or sinonasal symptoms.

### 2.3. Exclusion Criteria (Any of Following)

- Age < 18.
- Pregnancy, nursing.
- Medically compromised (ASA score > 1).
- Laryngological non-surgical treatment 3 months prior to RCT or/and during the course of RCT or/and in the period of observation (nasal drops, antibiotics).
- Laryngological/maxillofacial surgical treatment 1 year prior to RCT or/and in the period of observation.
- Endodontic surgical treatment (e.g., resection, radectomy, and extraction) in the period of observation.
- Systemic antibiotic administration during the course of endodontic treatment or/and in the period of observation.
- Trauma of the tooth that occurred during the observation period.
- Endo-perio pathology (PPD ≥ 3).
- Bone remodeling medications.

All treatments were performed between 2016 and 2024 by the same operator–specialist in endodontics (BC) at the same clinic with the same equipment and method, and according to the same treatment protocol over one or two appointments.

### 2.4. Treatment Protocol

Infiltration anesthesia was performed with 1 amp. of 4% articaine with 1:200,000 adrenaline both buccally and palatally (Septanest 1:200, Septodont, Saint-Maur-des-Fossés, France) and the operating field was isolated with a rubber dam (Hygenic Dental Dam Kit, Coltene/Whaledent, Altstätten, Switzerland). Afterwards, a dental operating microscope (DOM) was applied for every treatment (Leica M320, Leica Microsystems, Wetzlar, Germany). After access preparation, a glide-path was obtained in all canals using ISO 08 and 10 C-pilots (VDW, Frankfurt am Main, Germany) with a working length assessment with a Raypex 6 electronic apex locator (VDW, Germany). Chemo-mechanical preparation was performed with H-files (VDW, Germany) with a step-back technique up to a size of 20.02, followed by Endostar E3 rotary instruments (PolDent, Warsaw, Poland) with a crown-down technique up to an ISO size of 30.04 and MTwo rotary file ISO of 35.04, 40.04, or 45.04 (depending on the size of IAF) (VDW, Germany) as an MAF. If the MAF size was bigger than 45.04 or there was direct communication with the maxillary sinus, the apex was closed with 4 mm of an MTA plug (BioMTA, Cerkamed, Stalowa Wola, Poland). If a tooth was treated in two visits, canals were dried with sterile paper points (MetaBiomed, Cheongju-si, Republic of Korea); a sterile cotton pellet was applied on root canal orifices and the tooth was filled with a glassionomer temporary filling (Fuji IX, GC, Tokyo, Japan). The second visit was then scheduled for a period of a week. During all mechanical preparation, all canals were copiously irrigated with 40 mL of 5.25% sodium hypochlorite (NaOCl) (Chloraxid, Cerkamed, Poland), and after MAF preparation the final irrigation protocol was performed in all canals: 5.25% NaOCl in an amount of 5 mL/canal, 40% citric acid (CA) in an amount of 5 mL/canal, and in part of treatments a final flush with 2% chlorhexidine 5 mL/canal with manual dynamic irrigation (MDI) for additional antimicrobial effect. All canals were dried with sterile paper points (MetaBiomed, Republic of Korea) and obturated with a vertical compaction of warm guttapercha (GP) with the continuous wave of condensation (CWC) technique, using 35.04 master apical cones (MACs) (MetaBiomed, Republic of Korea) coated in a small amount of AHplus sealer (Dentsply Sirona, Charlotte, NC, USA), followed by a back-fill of 200 °C heated guttapercha with a Super Endo B&L system (B&L Biotech, Fairfax, VA, USA). A post-op intraoral radiograph (RVG) was taken to asses the direct post-operative effect of the treatment. Patients were instructed to return at 6 months, 1 year, or in the case of a flare-up. Informed consent was obtained from all patients involved in the study.

### 2.5. Outcome Assessment

CBCT scans were analyzed by two observers—a specialist in endodontics (BC) and a specialist in maxillofacial surgery (PS). MSEO was defined as the presence of periapical periosteitis (PAO) or/and periapical mucositis (PAM) adjacent to a treated tooth with coexisting pulp necrosis/an apical periodontitis (AP) lesion in its apical area. PAM was diagnosed when the thickness of antral mucosa measured on the sagittal, coronal, or axial cross-section of pre-op CBCT scans was >2 mm. The size of an AP lesion before the treatment was measured as the biggest linear measurement of the diameter of the lesion on a sagittal, coronal, or axial cross-section in CBCT scans, and on the basis of this measurement the CBCT PAI index, developed by Estrela et al. [12], was assessed. The same method and scale were used to assess AP size in control CBCT. Two additional variables—expansion and destruction of the sinus floor bone—were noted. Complete healing was diagnosed when CBCT PAI = 0 coexisted with a PAM < 2 mm in control CBCT.

Medical records were analyzed for inclusion and exclusion criteria and n = 114 teeth in 114 subjects entered the study. Medical records were then analyzed for fifteen factors that might have influenced treatment outcome: age, type of tooth treated, size of periapical lesion before RCT in the CBCT PAI scale, size of MT before RCT, exacerbation (upon admission/between treatment visits/after the treatment), number of endodontic interventions in a treated tooth (primary treatment, first retreatment, and second and next retreatment), number of visits in RCT (single- or two-visit RCT), time of observation, application of CHX in the final irrigation protocol, the presence of exudate from canals, apex MTA closure, expansion of the sinus floor bone, destruction of the sinus floor bone, and the presence of fistula. The observers were blinded to the factors studied.

### 2.6. Observer Calibration

To ensure the repeatability of CBCT measurements, intra- and inter-observer reproducibility were calculated [13]. The linear measurements scaled in the CBCT PAI index for an image set of randomly selected patients from clinic archives were recorded once per week for 5 weeks for both observers (BC and PS) in order to assess intra-observer reproducibility. In addition, each observer was calibrated with the same set of radiographs and intra-observer reproducibility was determined with an intraclass correlation coefficient of type 2 (according to Shrout and Fleiss) [14].

### 2.7. Statistical Analysis

The chi-squared test (with Yates correction for 2 × 2 tables) or Fisher’s exact test (in case of low expected values) were used for comparisons of healing between groups. The significance level was set to 0.05. All the analyses were conducted in R software, version 4.5.1 [15]. Multiple logistic regression was employed to model the potential impact of predictors on a dichotomous variable. ORs (odds ratios), alongside the 95% confidence intervals, were presented. Variables that showed significant univariate impact on dependent variables were included in the multiple logistic regression model.

## 3. Results

### 3.1. Subjects Characteristic

A total number of 114 teeth in 114 subjects entered the study, 53 female and 61 male patients (46.49% and 53.51%, respectively). The mean age of the subjects was 45.56 ± 13.25 years. In 93.86% of the subjects (n = 107), PAO and PAM symptoms were coexisting. The rate of the complete healing of MSEO after RCT was 76.32% (n = 87). The rate of the complete healing of a periapical lesion (CBCT PAI = 0) was 88.60% (n = 101). When PAO and PAM symptoms were analyzed separately, the resolution was 88.60% (n = 101) and 80.70% (n = 92), respectively. The teeth that were most frequently associated with MSEO were the first upper molar (52.63%), followed by the second upper molar (25.44%) and second upper premolar (14.04%). Most of the subjects were treated with two-visit RCT (61.40%, n = 70) and single-visit RCT was performed in 38.60% of the patients (n = 44). The most common size of the AP associated with MSEO was 4–8 mm (CBCT PAI = 4) (43.86% (n = 50)), and the mean diameter of periapical radiolucency associated with MSEO was 6.72 ± 4.89 mm. In 28.95% (n = 33) of the subjects, the size of AP exceeded 8 mm in diameter (CBCT PAI = 5). Expansion of the sinus floor bone was detected in 50.88% of the patients (n = 58), with destruction of the sinus floor present in 45.61% of the subjects (n = 52). In 39.47% of the patients (n = 45), the sinus was severely affected, with MT greater than 10 mm. The highest MSEO healing rate was observed 13–24 months after the treatment (49.12%). In most of the treatments (75.44%), CHX was applied as a final flush in the irrigation protocol. Exudate in canals during the treatment was present in 20.18% of the cases (n = 23), fistula was present in 22.81% of the subjects (n = 26), and flare-up at any stage of RCT was detected in 8.77% of patients (n = 10). In 10.53% (n = 12) of the teeth, one or more canals were closed with an MTA plug. In most of the cases, RCT was a first retreatment (75.44%, n = 86). Full subject characteristics are presented in Table 1.

### 3.2. Observer Agreement

The inter- and intra-rater reliability for both observers (BC and PS) calculated with ICC2 are presented in Table 2 and Table 3.

### 3.3. Factors Influencing the Healing of MSEO After Non-Surgical RCT

**Figure 1:**
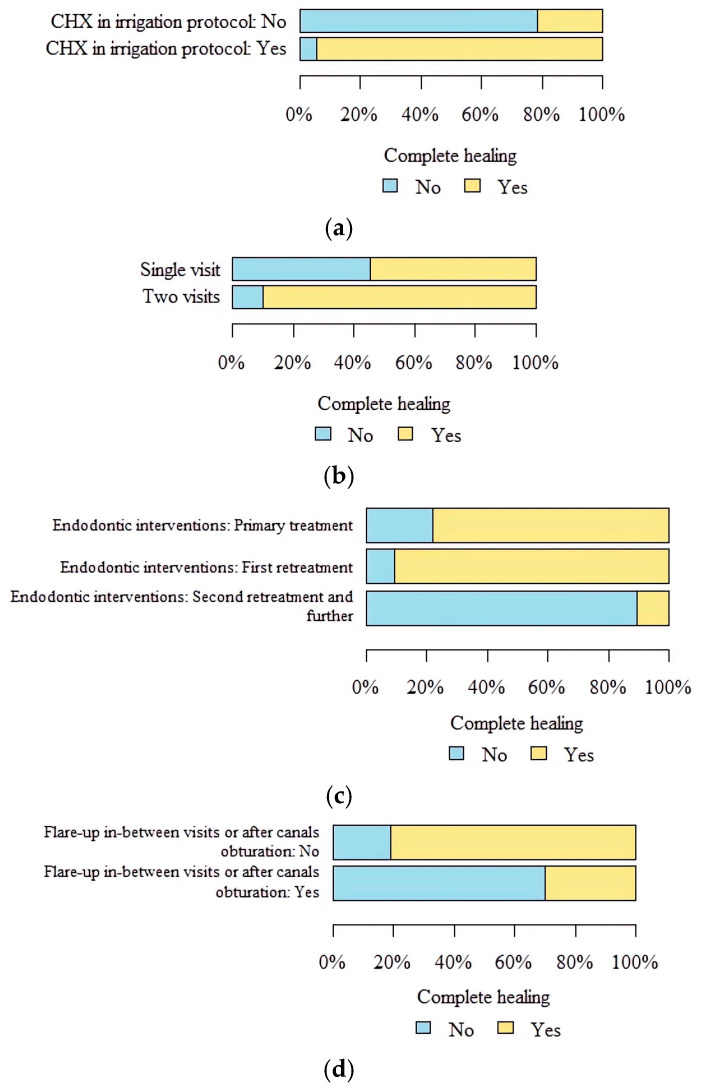
Factors that significantly influenced the healing of MSEO: (**a**) application of CHX in a root canal irrigation protocol; (**b**) RCT treatment mode (single-visit RCT vs. two-visit RCT); (**c**) number of endodontic interventions in a treated tooth; and (**d**) exacerbation at any stage of RCT.

Fifteen factors were chosen for their possible influence on the healing of MSEO, with the results listed in Table 4 and Table 5. *p* values below 0.05 indicate statistically significant differences between groups. Variables that showed significant univariate impact on dependent variables were included in the multiple logistic regression model (Table 5). MSEO was more likely to heal when a tooth was treated in two appointments than when a single-visit RCT was performed (*p* = 0.012). The number of endodontic interventions significantly influenced the healing of MSEO, as it was less likely to heal when there was more than one endodontic retreatment in a tooth (*p* = 0.01). The healing of MSEO was higher when CHX was applied in a final irrigation protocol (*p* = 0.022) and was less likely when there was a flare-up in-between visits or after obturation of the canals (*p* = 0.002). Two more factors might have influenced MSEO healing: age of the patient (*p* = 0.041) and time of observation (*p* = 0.02); however, these were not confirmed when the multiple logistic regression model was applied. The coexistence of both PAO and PAM symptoms did not significantly influence the healing of maxillary sinusitis. The healing of MSEO was not dependent on the size of the AP lesion and mucosa thickness before the treatment. Factors like tooth morphology, destruction or expansion of the sinus floor bone, fistula presence, or MTA apex closure did not significantly influence the healing. Factors that significantly influenced the healing of MSEO are presented in Figure 1.

## 4. Discussion

Although CBCT-based evidence on the resolution of maxillary sinusitis after non-surgical endodontic treatment has been provided in the past and there have been attempts to indicate factors that may influence the healing of MSEO after RCT [8], the literature still lacks studies with larger numbers of subjects [8,16]. Moreover, in past studies, RCTs were performed by multiple endodontic operators with different levels of experience and practical skills, which implies operator bias [8]. To decrease these at the stage of performing RCT, all treatments in our study were performed by the same operator, a specialist in endodontics (BC), according to the same, standardized treatment protocol. Moreover, our inclusion and exclusion criteria were more strict than in previous studies [8], to ensure that there were no internal and external factors that might have influenced the course of maxillary sinusitis healing.

Different to previous studies that suggested the influence of the features of periapical lesion [16], our study showed no association between AP size prior to RCT and the rate of healing of MSEO. Despite the fact that it was proven that molars are 11 times more likely than premolars to cause MSEO when both teeth are present [17], the type of treated tooth had no significant influence on maxillary sinusitis healing.

The discussion on whether single-visit or multiple-visit RCT is more effective in terms of AP healing is as old as endodontics has been a science. Currently, it can be stated that there is no evidence that one treatment regimen is more effective than the other [18]; however, within the limitations of our study, the rate of healing of MSEO was significantly higher when a tooth was treated in two appointments. This can be explained by the fact that the time of contact between endodontic space and antiseptics that are applied to it during two-visit RCT is longer than when single-visit RCT is performed. Moreover, the volume of NaOCl used in the chemical preparation of root canals is bigger in two-visit RCT. Additionally, the treatment protocol in our study was designed in a way to eliminate the risk of the reinfection of endodontic space in-between visits, which is the weakest point of all multi-visit RCTs and a factor that significantly decreases the rate of AP healing [19].

The application of CHX in the root canal irrigation protocol was reported to improve the outcome of non-surgical RCT in many previous studies [20,21], and because MSEO was proven to be caused by oral pathogens [22] we hypothesized that the application of CHX as a final flush can improve the healing of MSEO after RCT. Indeed, the healing rate of maxillary sinusitis was higher in the group where 2% CHX was introduced in the final irrigation protocol.

The presence of fistula was chosen as factor for its possible influence on MSEO healing, as it is thought to be a bad prognostic factor for RCT outcomes [16]; however, we did not confirm its influence on MSEO resolution. The number of endodontic interventions significantly influenced the healing of MSEO, as it was less likely to heal when there was more than one endodontic retreatment in a tooth (*p* = 0.01).

Moreover, the healing of MSEO was less likely when there was a flare-up in-between visits or after obturation of the canals (*p* = 0.002), which confirms that exacerbation in the course of this disease at any stage of the treatment is also a bad prognostic factor for MSEO healing.

## 5. Conclusions

This study showed a high rate of MSEO resolution (76.32%) after non-surgical RCT. Within the limitations of this retrospective study, we indicated 4 out of 15 factors that had a significant influence on the healing. The application of CHX in the final irrigation protocol and a two-visit endodontic treatment mode significantly improved the healing of MSEO. MSEO was less likely to heal when there were more than two endodontic interventions in a tooth and when there was the presence of a flare-up at any stage of the treatment. Those factors should be taken into consideration in obtaining treatment protocols for dental-induced sinusitis and the better assessment of the possible success of this non-invasive treatment approach.

## Figures and Tables

**Table 1:** Subject characteristics.

Parameter		Total (N = 114)
Age [years]	Mean (SD)	45.56 (13.25)
	Median (quartiles)	45 (37–52.75)
	Range	20–74
	n	114
Sex	Female	53 (46.49%)
	Male	61 (53.51%)
Complete healing (PAO + PAM resolution)	No	27 (23.68%)
	Yes	87 (76.32%)
PAO resolution	No	13 (11.40%)
	Yes	101 (88.60%)
PAM resolution	No	22 (19.30%)
	Yes	92 (80.70%)
Tooth type	First upper premolar	8 (7.02%)
	Second upper premolar	16 (14.04%)
	First upper molar	60 (52.63%)
	Second upper molar	29 (25.44%)
	Third upper molar	1 (0.88%)
Pre-op size of apical lesion [mm]	Mean (SD)	6.72 (4.89)
	Median (quartiles)	5.9 (3.82–8.38)
	Range	0–30.6
	n	114
Pre-op CBCT PAI	0	7 (6.14%)
	1	1 (0.88%)
	2	1 (0.88%)
	3	22 (19.30%)
	4	50 (43.86%)
	5	33 (28.95%)
Pre-op thickness of mucosa	Up to 5 mm	30 (26.32%)
	5–10 mm	39 (34.21%)
	Over 10 mm	45 (39.47%)
Coexistence of periapical lesion and thickness of mucosa	No	7 (6.14%)
	Yes	107 (93.86%)
Expansion of the sinus floor bone before treatment	No	56 (49.12%)
	Yes	58 (50.88%)
Destruction of the sinus floor bone before treatment	No	62 (54.39%)
	Yes	52 (45.61%)
Time of observation [months]	6–12 months	42 (36.84%)
	13–24 months	56 (49.12%)
	Over 24 months	16 (14.04%)
Post-op CBCT PAI	0	101 (88.60%)
	1	0 (0.00%)
	2	6 (5.26%)
	3	3 (2.63%)
	4	2 (1.75%)
	5	2 (1.75%)
Post-op thickness of mucosa [mm]	Mean (SD)	1.51 (3.3)
	Median (quartiles)	0 (0–1.2)
	Range	0–15.6
	n	114
Treatment mode	Single visit	44 (38.60%)
	Two visits	70 (61.40%)
CHX in irrigation protocol	No	28 (24.56%)
	Yes	86 (75.44%)
Apex MTA closure	No	102 (89.47%)
	Yes	12 (10.53%)
Exudate in canals during treatment	No	91 (79.82%)
	Yes	23 (20.18%)
Presence of fistula	No	88 (77.19%)
	Yes	26 (22.81%)
Flare-up in-between visits or after canal obturation	No	104 (91.23%)
	Yes	10 (8.77%)
Number of endodontic interventions	Primary treatment	9 (7.89%)
	First retreatment	86 (75.44%)
	Second retreatment and further	19 (16.67%)

**Table 2:** Inter-rater reliability.

ICC	95% CI		Agreement (Koo and Li)
0.826	0.617	0.927	Good

**Table 3:** Intra-rater reliability.

Parameter	ICC	95% CI		Agreement (Koo and Li)
Observer 1 (BC)	0.868	0.595	0.990	Good
Observer 2 (PS)	0.844	0.540	0.988	Good

**Table 4:** Factors influencing the healing of MSEO.

Parameter	Group	Complete Healing		*p*
		No	Yes	
Age	Up to 35 years (N = 24)	1 (4.17%)	23 (95.83%)	*p* = 0.041 *
	36–45 years (N = 34)	9 (26.47%)	25 (73.53%)	
	46–55 years (N = 33)	8 (24.24%)	25 (75.76%)	
	Over 55 years (N = 23)	9 (39.13%)	14 (60.87%)	
Tooth morphology	Tooth 4 (N = 8)	0 (0.00%)	8 (100.00%)	*p* = 0.194
	Tooth 5 (N = 16)	2 (12.50%)	14 (87.50%)	
	Tooth 6 (N = 60)	18 (30.00%)	42 (70.00%)	
	Tooth 7 or 8 (N = 30)	7 (23.33%)	23 (76.67%)	
Observation time	6–12 months (N = 42)	4 (9.52%)	38 (90.48%)	*p* = 0.02 *
	13–24 months (N = 56)	18 (32.14%)	38 (67.86%)	
	Over 24 months (N = 16)	5 (31.25%)	11 (68.75%)	
Size of the lesion (in CBCT PAI scale)	0–2 (N = 9)	4 (44.44%)	5 (55.56%)	*p* = 0.05
	3 (N = 22)	5 (22.73%)	17 (77.27%)	
	4 (N = 50)	15 (30.00%)	35 (70.00%)	
	5 (N = 33)	3 (9.09%)	30 (90.91%)	
Coexistence of PAO and PAM symptoms	No (N = 7)	3 (42.86%)	4 (57.14%)	*p* = 0.353
	Yes (N = 107)	24 (22.43%)	83 (77.57%)	
Expansion of the sinus floor bone	No (N = 56)	14 (25.00%)	42 (75.00%)	*p* = 0.917
	Yes (N = 58)	13 (22.41%)	45 (77.59%)	
Destruction of the sinus floor bone	No (N = 62)	15 (24.19%)	47 (75.81%)	*p* = 1
	Yes (N = 52)	12 (23.08%)	40 (76.92%)	
Pre-op thickness of mucosa (PAM size)	Up to 5 mm (N = 30)	3 (10.00%)	27 (90.00%)	*p* = 0.121
	5–10 mm (N = 39)	11 (28.21%)	28 (71.79%)	
	Over 10 mm (N = 45)	13 (28.89%)	32 (71.11%)	
Treatment mode	Single visit (N = 44)	20 (45.45%)	24 (54.55%)	*p* < 0.001 *
	Two visits (N = 70)	7 (10.00%)	63 (90.00%)	
Number of endodontic interventions	Primary treatment (N = 9)	2 (22.22%)	7 (77.78%)	*p* < 0.001 *
	First retreatment (N = 86)	8 (9.30%)	78 (90.70%)	
	Second retreatment and further (N = 19)	17 (89.47%)	2 (10.53%)	
CHX in irrigation protocol	No (N = 28)	22 (78.57%)	6 (21.43%)	*p* < 0.001 *
	Yes (N = 86)	5 (5.81%)	81 (94.19%)	
Exudate in canals during treatment	No (N = 91)	20 (21.98%)	71 (78.02%)	*p* = 0.563
	Yes (N = 23)	7 (30.43%)	16 (69.57%)	
Presence of fistula	No (N = 88)	19 (21.59%)	69 (78.41%)	*p* = 0.481
	Yes (N = 26)	8 (30.77%)	18 (69.23%)	
Flare-up in-between visits or after canal obturation	No (N = 104)	20 (19.23%)	84 (80.77%)	*p* = 0.002 *
	Yes (N = 10)	7 (70.00%)	3 (30.00%)	
Apex MTA closure	No (N = 102)	26 (25.49%)	76 (74.51%)	*p* = 0.288
	Yes (N = 12)	1 (8.33%)	11 (91.67%)	

*—statistically significant (*p* < 0.05); *p*—chi-squared or Fisher’s exact test.

**Table 5:** Factors influencing the healing of MSEO analyzed with multiple logistic regression.

Trait		N	n	OR	95%CI		*p*
Age [years]		-	-	0.967	0.862	1.083	0.559
Time of observation	6–12 months	42	38	1	ref.		
	13–24 months	56	38	0.351	0.016	7.54	0.504
	Over 24 months	16	11	0.158	0.003	8.787	0.368
Pre-op CBCT PAI	0–2	9	5	1	ref.		
	3	22	17	159.443	0.492	51,696.69	0.086
	4	50	35	6.351	0.234	172.431	0.272
	5	33	30	405.151	0.919	178,664.835	0.053
Treatment	Single visit	44	24	1	ref.		
	Two visits	70	63	81.94	2.635	2548.131	0.012 *
CHX in irrigation protocol	No	28	6	1	ref.		
	Yes	86	81	29.311	1.639	524.232	0.022 *
Endodontic interventions	Primary treatment	9	7	1	ref.		
	First retreatment	86	78	0.063	0.001	12.201	0.304
	Second retreatment and further	19	2	0.001	0.0001	0.133	0.01 *

*p*—multiple logistic regression; N—group size; n—cases of complete healing; and *—statistically significant (*p* < 0.05).

## Data Availability

Data are contained within the article.

## Abbreviations

The following abbreviations are used in this manuscript:

MSDO	Maxillary sinusitis of dental origin
AAE	American Association of Endodontists
MSEO	Maxillary sinusitis of endodontic origin
RCT	Root canal treatment
ESS	Endoscopic sinus surgery
CBCT	Cone beam computed tomography
ASA	American Society of Anesthesiologists
PAO	Periapical osteoperiostitis
PAM	Periapical mucositis
PPD	Probing pocket depth
DOM	Dental operating microscope
ISO	International Organization for Standardization
MAF	Master apical file
MTA	Mineral trioxide aggregate
NaOCl	Sodium hypochlorite
CA	Citric acid
CHX	Chlorhexidine
MDI	Manual dynamic irrigation
GP	Guttapercha
CWC	Continuous wave of condensation
RVG	Radiograph
AP	Apical periodontitis
MT	Mucosa thickness
